# Nitric oxide (NO) involved in Cd tolerance in NHX1 transgenic duckweed during Cd stress

**DOI:** 10.1080/15592324.2022.2065114

**Published:** 2022-04-26

**Authors:** Qiuting Ren, Na Li, Ruxin Liu, Xu Ma, Jinge Sun, Jianyao Zeng, Qingqing Li, Mingwei Wang, Xinglin Chen, Xiaoyu Wu, Lin Yang

**Affiliations:** a Tianjin Key Laboratory of Animal and Plant Resistance, College of Life Sciences, Tianjin Normal University, Tianjin, Xiqing, China; b School of Basic Medical Sciences, Fudan University, Shanghai, Yangpu, China; c Center for Infection and Immunity Studies, School of Medicine Sun Yat-san University, Shanghai, China; d School of Medicine, Shanghai University, Shanghai, Baoshan, China

**Keywords:** Cadmium stress, NHX1 transgenic duckweed, NO, gene expression

## Abstract

Anthropogenic activities cause heavy metal pollution, such as cadmium (Cd). Na^+^/H^+^ antiporter (NHX1) transgenic duckweed showed Cd tolerance in our previous study, and the signal mechanism needs to be explored. As an important signal molecule, nitric oxide (NO) is involved in a number of functions under abiotic stress response. This study analyzed the levels of endogenous NO in wild-type (WT) duckweed and NHX1 duckweed under Cd treatment. The results showed that after 24 h Cd treatment, the endogenous NO level of WT duckweed decreased, which was significantly lower than that in NHX1 duckweed. Studies have proved that NHX1 influences pH. The level of NO in this study has been investigated at different pH. The NO level was the highest in the duckweed cultured with pH 5.3. Nitrate reductase gene expression was down-regulated and NO synthesis was decreased under Cd stress in WT duckweed. This study showed that NO level has been modified in NHX1 duckweed, which could be influcened by pH.

## 1. Introduction

Cadmium (Cd) is a heavy metal toxin listed as one of the most dangerous materials.^1^ Cd is one of the main water pollutants because of its strong toxicity, strong migration, and extensive pollution.^2^ Cd pollution in water and soil caused the accumulation in plants, which might enter the human body through the food chain, and do harm to human health. Low concentrations of cadmium can pose a threat to plants and animals.^2^ Cd causes damage to various physiological and biochemical activities of plants, such as causing leaf yellowing and affecting plant photosynthesis.^3^ Cd also impairs plant cell division and membrane activity. Cadmium can cause symptoms such as slow plant growth, stunted plant and leaf chlorosis. Cadmium stress can up-regulate heat shock protein genes in plant roots, and increase the gene expression of cytochrome secondary metabolism in plant leaves under cadmium stress, thus reducing photosynthesis.^4^ Therefore, it is important to decrease the Cd accumulation in plant and study the signal responds during Cd stress.

Nitric oxide (NO) is a vital part in the regulation of important physiological processes in plants and animals. As an indispensable signaling molecule, NO can help regulate plant life activities. Under drought stress, NO can activate the reactive oxygen scavenging enzyme system, enhance the antioxidant system,^5^ and make plants produce drought tolerance mechanism.^6^ Studies have shown that exogenous application of NO can increase the dry weight^7^ of seedlings, such as maize under salt stress.^8^ During the process of heavy metal stress, endogenous NO accumulated under the action of heavy metal stress (HMs). After 48 hours of Cd treatment, the level of endogenous NO decreased significantly in the root of Barrel medic.^9^ Duckweed is a simple aquatic plant, which has the characteristics of rapid propagation, convenient cultivation, and wide distribution. It is considered to be an indicator plant for environmental toxicity test. However, the NO signal response in duckweeds has not yet been studied.

In our previous studies, we have successfully studied the NHX1 transgenic duckweed (OE) and further proved that NHX1 can improve the tolerance of duckweed to Cd^2+^, and the accumulation of Cd in duckweed OE under Cd stress is less than it in WT.^10^ Na^+^/ H^+^ transgenic protein (NHX1) plays a vital role in adjusting endosomal pH, salt-resistance and Na^+^-k^+^ homeostasis.^11^ In further experiments, we found that during Cd treatment, NHX1 duckweed promoted the outflow of Cd^2+^ and increased the inflow of H^+^, thus changing the pH, which triggered the outbreak of H_2_O_2_ and further the expression of SOD, POD, or other enzymes can be regulated. This shows that under short-term Cd^2+^ shocks, the combined action of NHX1, ROS, Cd^2+^ ionic current, and pH can improve the resistance of duckweed to Cd treatment.^12^ The NO signal response to Cd in NHX1 duckweed is still to be investigated.

In our experiment, we will specifically study the relationship between Cd stress resistance and endogenous NO in NHX1 transgenic duckweed.

## 2. Materials and methods

### 2.1 Cultivate of duckweed

Duckweed (*Lemna turionifera* 5511) was taken from the Fengchan river of Tianjin. The duckweed was cultured in the liquid medium Datko, which contains 0.4 mM MgSO_4_ · 7H_2_O, 1.4 mM Ca (NO_3_)_2_ · 4H_2_O, 1.1 mM KNO_3_, 0.4 mM KH_2_PO_4_, 0.4 mM Mg (NO_3_)_2_ · 6H_2_O, 55 µM CaCl_2_ · 2H_2_O, 55 µM KCl, 6.2 µM Na_2_MoO_4_ · 2H_2_O, 71 µM H_2_BO_3_, 30 µM K_2_H_2_EDTA·2H_2_O, 56.7 µM FeNH_4_EDTA, 13.8 µM MnCl_2_ · 4H_2_O, 2.8 µM ZnNa_2_EDTA·4H_2_O, 4.8 µM CoSO_4_ · 7H_2_O, 18.6 µM Na_2_EDTA·2H_2_O, with their pH adjusted to 5.8 ± 0.1.^10^ Duckweed was cultured at 24 ± 1°C with the photoperiod set as 16 hours a day and the light intensity was 95 ± 5 μmol m^−2^·s,^−15^ which was subcultured once a week. The *Na^+^/H^+^ Antiporter* (NHX1) duckweed was obtained and identified in our former studies.^10^

### 2.2 Detection of endogenous NO

Duckweeds were treated with 50 μM CdCl_2_ in the liquid media supplemented together with or without 0.5 mM amiloride for 2 and 24 hours, 16 hours light, and 8 hours darkness. Each treatment grew more than 30 fronds. Specific NO fluorescence probe DAF-FM^TM^ (4-amino-5-methylamino-2’,7’-difluororescein; Cat. No. D-23841, Invitrogen) was used to monitor endogenous NO levels through the fluorescence microscope (Leica DFC450C, DM5000, Berlin, Germany) and the results were recorded in the form of pictures. According to the instructions of DAF-FM^TM^ user guide, 5 mM stock solution of DAF-FM™ diacetate was made by dissolving the 1 mg DAF-FM™ in 0.4 mL of DMSO, and then the stock solution was diluted by 0.1 M phosphate buffer (pH 7.0) to 10 μM as a dye working solution. Put the duckweed root sample into a 1.5 ml EP tube with 300 μl PBS. Suck out PBS, then add 300 μl fixed solution, and incubated for 15 minutes. The fixed solution was sucked out, 300 μl PBS was added, and the root samples were washed three times by PBS. Suck out PBS, and the duckweed root sample was immersed in 5 μl DAF-FM working solution for 30 minutes under the dark condition of 37°C, then the dye was sucked out and washed with 300 μl PBS for three times. Finally, five treated rhizoid samples were randomly selected and made into plates by PBS. The endogenous NO levels were monitored and photographed by the fluorescence microscope.

### 2.3 Measure changes in related genes

The duckweed samples were frozen with liquid nitrogen and sent to Novogene (Chaoyang, Beijing) for the gene sequencing and expression. We compared the wild-type (WT) duckweed or NHX1 duckweed with or without 50 μM CdCl_2_ stressed WT for 24 h, and 1.5 µg RNA per sample were used for transcriptome sequencing. Select gene annotation in the following authoritative database: Swiss-Prot (Annotated protein sequence database), KOG/COG (Clusters of Orthologous Groups of proteins), GO (Gene Ontology), Pfam (Protein family), Nt (NCBI non-redundant nucleotide sequences), KO (KEGG Ortholog database) and Nr (NCBI non-redundant protein sequences). Evaluate the gene expression level of samples by the RNA-Seq by Expectation Maximization (RSEM) (Hoffmann *et al*., ^16^2010) in the experiment. The genes associated with NO were selected and their expression levels were analyzed.

### 2.4 Calcium on the fronds and roots of duckweed observed by SEM and EDX analysis

The WT and OE duckweeds after 2 h CdCl_2_ stress were treated by dessication and spraying gold. Then morphologies of frond and roots were measured by field emission technique scanning electronic microscopy (SEM, Nova Nano SEM 230). And the Ca element level was determined by energy-dispersive X-ray spectrometer (EDX, Genesis APEX, Genesis Apollo 10).

### 2.5 Data statistics and analysis

All the data measured in this experiment were analyzed by SPSS software (IBM SPSS Statistics, version 20), and the variables were tested by the independent sample T-test. The error bars are represented by the standard deviation (±SD). The significant differences are indicated by asterisks (* P < .05, ** P < .01). Repeated all experiments at least three times, with each parallel group containing more than six groups, each containing 50–120 leaves.

## 3. Result

### 3.1 Transcriptional analysis of NO pathway in duckweed under Cd stress

**Figure 1.**
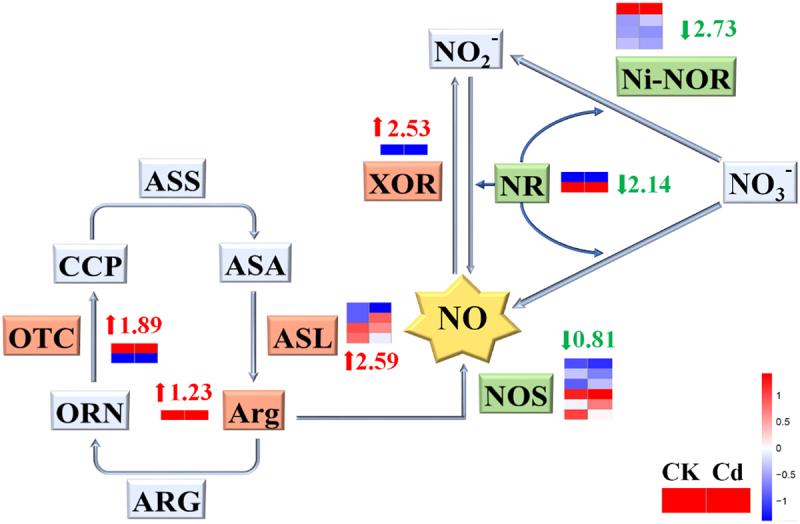
Differences of NO metabolism mechanism between CK group and Cd group.Red arrow is up-regulated, and green arrow is down-regulated. CK group represent wild-type (WT) duckweed, and Cd group represent wild-type (WT) duckweed under 50 μM Cd stress.The color in this figure legend from red to blue, which means log_10_ (FPKM+1) from high to low. Red means high expression, blue means low expression.

The expression of genes involved in nitric oxide metabolism pathway was explored in the duckweed treated without (CK) or with Cd for 24 h in Nitric oxide is not only a product of the urea cycle, but also a product of nitrate ions showed as Figure 1. The metabolic pathways of nitric oxide and arginine are clearly expressed. Ornithine (ORN), produced from the urea cycle pathway, can produce cyclic citrullinated peptide (CCP). Ornithine transcarbamylase (OTC) is the key enzyme in the formation of CCP from ORN, which has raised 1.89 log_2_ Fold Change. Then, conversion of CCP to argininosuccinic acid (ASA) by argininosuccinate synthetase (ASS). ASA is the precursor substance of arginine (Arg). Argininosuccinate lyase (ASL) is the key enzyme in this process, which has raised 2.59 log_2_ Fold Change. Arginase (ARG) is a key enzyme that converts Arg to ORN, which has raised 1.23 log_2_ Fold Change. Nitric Oxide Synthase (NOS) participates in the synthesis of NO from Arg, which has fallen by 0.81 log_2_ Fold Change. Xanthine oxidoreductase (XOR) participates in the synthesis of NO_2_^−^ from NO, which has raised 2.53 log_2_ Fold Change. Ni-NOR is the key enzyme in the formation of NO_2_^−^ from NO_3_^−^, which has fallen by 2.73 log2 Fold Change. While nitrate reductase (NR) is the key enzyme that converts NO_3_^−^ and NO into NO_2_^−^, which has fallen by 2.14 log_2_ Fold Change. These results revealed that the addition of Cd can reduce the synthesis of NO to a certain extent, thus affecting the content of NO.

### 3.2 NO accumulation is accompanied with increased Cd treat time in NHX1 transgenic duckweed

**Figure 2.**
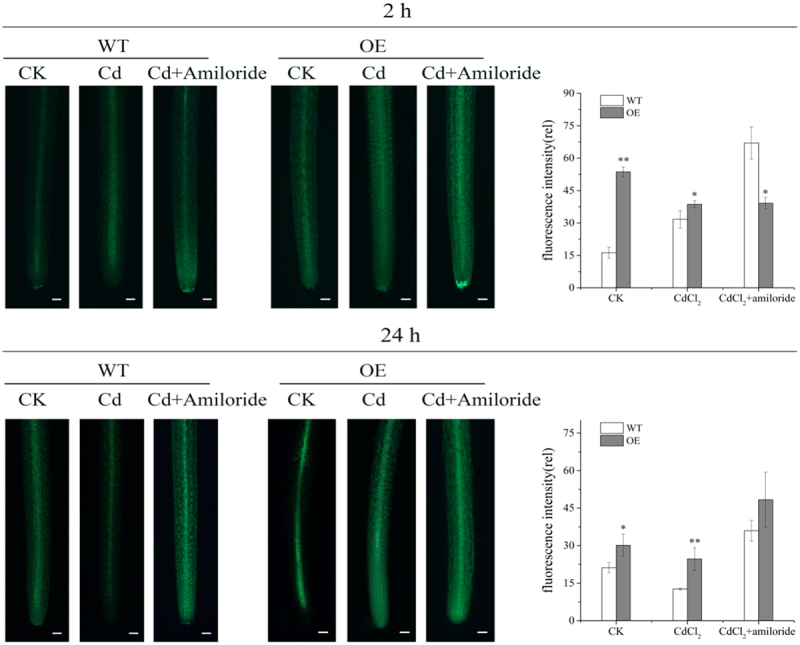
NO detection in OE rhizoid and WT one. Treated duckweed with 50 μM CdCl_2_ together with or without 0.5 mM amiloride for 2 hours (a) and 24 hours (b), and stained the rhizoid with DAF-FM. Scale bar = 100 μm) relative NO fluorescence the density of the root tips. CK: Significant difference was determined by independent sample t-test and indicated by asterisks (*P < .05, **P < .01).

The NO signal, stained by NO specific fluorescence probe DAF-FM DA, was studied to investigate the involvement of NO during Cd stress in NHX1 duckweed (Figure 2). With 2 h CdCl_2_ (50 μM) shock, the NO level in WT was much lower than that in NHX1, about 0.5 times to the NO level in NHXI. However, with 24 h CdCl_2_ (50 μM) treatment, the NO level in WT duckweed declined. And the similar result was reported in Pea (*Pisum sativum* ‘Lincoln’), which was supplemented with 50 mM CdCl_2_ for 14 days (Rodríguez-Serrano *et al*., ^17^2006). Compared to that, the NO level in NHX1 was significantly higher than it in WT with 24 h CdCl_2_ treatment. With the addition of amiloride, the NO level in both WT and OE duckweed was enhanced significantly.

### 3.3 Changes of relative gene expression in NHX1 duckweed with Cd treatment

As showed in Table 1, the expression of nitrate reductase has been significantly reduced after Cd treatment in OE duckweed (Table 1). This result indicates that the metabolism of NO has been regulated by stress. Compared to WT duckweed, the expression level of nitrate reductase in OE duckweed was also significantly decreased after 24 h Cd stress, suggesting that the conversion of NO_2_^−^ to NO was significantly decreased in OE duckweed under Cd stress. This indicates that under Cd stress, NO synthesis from NO^11−^ in OE duckweed has been down-regulated than wild duckweed. (Table 2).

**Table 1.** Changes in gene expression levels related to nitrate reductase. (OE-Cd vs OE)

Description	Gene ID	OE_Cd_	WT_Cd_	log_2_Fold	pval	padj
		Readcount	Readcount	Change		
nitrate reductase (NADPH)	Cluster-7365.57428	3968.38	29159.25	−2.88	3.13E-133	4.74E-131
nitrate reductase (NADPH)	Cluster- 7365.56158	329.49	2716.49	−3.04	9.62E-37	3.44E-35

**Table 2.** Changes in gene expression levels related to nitrate reductase. (OE-Cd vs WT-Cd)

Description	Gene ID	OE_Cd_	WT_Cd_	log_2_Fold	pval	padj
		Readcount	Readcount	Change		
nitrate reductase	Cluster-7365.57428	4229.60	9056.14	−1.10	1.95E-10	3.40E-08
catalase isozyme 1	Cluster-7365.59922	69.68	30.78	1.18	1.44E-05	8.22E-04
isocitrate dehydrogenase (NAD) subunit 1	Cluster-7365.64585	199.72	0.96	7.67	2.24E-23	1.74E-20
isocitrate dehydrogenase [NADP]	Cluster-7365.80877	14.78	0.23	5.64	8.36E-04	2.47E-02
Isocitrate dehydrogenase [NAD] regulatory subunit 1	Cluster-7365.47854	23.60	0.00	7.03	1.87E-03	4.69E-02
NADH dehydrogenase (ubiquinone) 1 beta subcomplex subunit 9	Cluster-7365.42787	1646.39	597.38	1.46	7.47E-05	3.35E-03
acyl-coenzyme A oxidase 3	Cluster-7365.23419	33.09	0.00	7.52	1.12E-08	1.36E-06
acyl-coenzyme A thioesterase 13	Cluster-7365.19158	52.83	21.73	1.28	2.73E-04	9.88E-03
1-aminocyclopropane-1-carboxylate synthase (ACS2)	Cluster-7365.799	106.38	37.21	1.51	3.68E-07	3.28E-05
mitogen-activated protein kinase kinase kinase 1 (MAPKKK 1)	Cluster-7365.78772	31.54	0.00	7.45	5.21E-08	5.51E-06

In previous studies, NO interacts with ROS to regulate colonization, cell death, and resistance processes.^18^Also, France et al. presented a model showing the role of NO and S-nitrosylation during drought stress signaling, and stress triggers ABA accumulation that in turn can induce the production of H_2_O_2_ leading to the generation of NO, resulting in stomatal closure via the activation of a MAPK pathway.^19^ Moreover, there was a feedback mechanism between ethylene and NO through the activity of ACC/ACO/ACS.^20^ In this study, the expression of key proteins participated in the signal pathway of NO interaction with ROS, ABA, and ethylene has been analyzed. Shown as in Table 3, catalase isozyme 1 raised 1.18 log_2_ Fold Change. The expression of isocitrate dehydrogenase (NAD) subunit 1, isocitrate dehydrogenase [NADP], and Isocitrate dehydrogenase [NAD] regulatory subunit 1 enhanced 7.67, 5.64 and 7.03 log_2_ Fold Change, respectively. The expression of 1-aminocyclopropane-1-carboxylate synthase (ACS2) has increased by 1.51 log_2_ Fold Change. Also, mitogen-activated protein kinase kinase kinase 1 (MAPKKK 1) has been up-regulated in NHX1 duckweed compared with WT duckweed during Cd stress.

**Table 3.** Changes in gene expression levels related to calmodulin. (OE-Cd vs WT-Cd)

Description	Gene ID	OE_Cd	WT_Cd	log_2_Fold	pval	padj
		Readcount	Readcount	Change		
calmodulin	Cluster-7365.17727	655.71	3274.73	−2.32	0.00	3.58E- 104
calmodulin	Cluster-172.0	0.00	12.77	−6.15	0.00	0.00
calmodulin	Cluster-7365.104603	13.15	38.49	−1.55	0.00	0.03
calmodulin	Cluster-117.0	0.00	6.80	−5.24	0.00	0.04
calmodulin	Cluster-7365.10784	18.39	0.00	6.68	0.00	0.05
calmodulin	Cluster-7365.88501	5.42	20.62	−1.93	0.00	0.05
calmodulin-binding transcription activator 2-like isoform X1 [Phoenix dactylifera]	Cluster-7365.17053	180.12	59.42	1.60	0.00	0.00
kinesin-like calmodulin-binding protein homolog isoform X1 [Nelumbo nucifera]	Cluster-7365.77511	121.22	46.46	1.38	0.00	0.01
IQ calmodulin-binding motif	Cluster-7365.93455	31.16	13.74	1.19	0.00	0.04
IQ calmodulin-binding motif	Cluster-7365.33198	23.53	1.22	4.26	0.00	0.04
calcium/calmodulin-dependent protein kinase	Cluster-7365.10784	18.39	0.00	6.68	0.00	0.05
calmodulin-binding receptor-like cytoplasmic kinase 2 [Ananas comosus]	Cluster-7365.88501	5.42	20.62	−1.93	0.00	0.05

### 3.4 NO accumulation is related to Ca^2+^ in NHX1 duckweed

**Figure 3.**
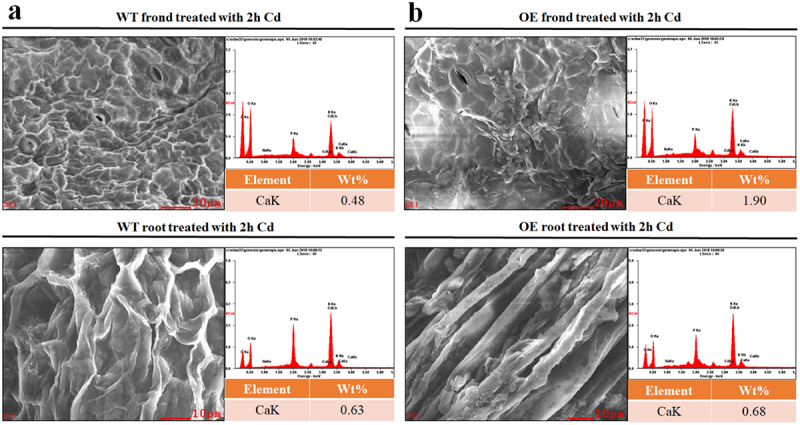
SEM images of the fronds and roots of WT (a) and OE (b) duckweed treated with Cd (50 μM) for 2 h.The OE duckweed represents NHX1 duckweed.

As can be seen from Table 3, the expression of calmodulin in OE duckweed changed compared to that in WT duckweed under Cd stress, and five genes were down-regulated and one gene up-regulated. However, the expressions of calmodulin-binding proteins have been up-regulated. For example, the expressions of calmodulin-binding transcription activator 2-like isoform X1 were enhanced by 1.60 and 1.38 log_2_ Fold. And the expression of calcium/calmodulin-dependent protein kinase was increased by 6.78 log_2_ Fold. To further study the link of Ca^2+^ and NO, the level of Ca^2+^ in OE and WT duckweed plants during Cd stress has been studied. The surface morphology and elemental composition of fronds and roots were analyzed by SEM and EDX. The results showed as Figure 3 indicated that the calcium content of the WT duckweed was lower than the NHX1 transgenic duckweed (OE). As to the fronds surface of the WT duckweed, the Ca^2+^ content was 0.48%, while the Ca^2+^ content of the NHX1 transgenic duckweed was 1.90%. And the roots surface of the WT duckweed, the Ca^2+^ content was 0.63%. While the Ca^2+^ content of the NHX1 transgenic duckweed was 0.68%.

### 3.5 NO accumulation is related to environmental pH during Cd treatment in NHX1 transgenic duckweed

**Figure 4.**
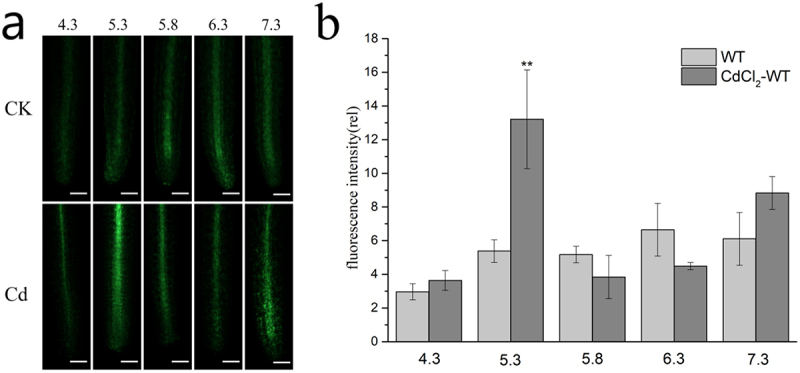
NO detection in WT duckweed. Duckweed was treated at different pH for 24 hours, and the rhizomes were stained with DAF-FM. Figure 2 shows the fluorescence intensity of duckweed treated with NO dye for 24 hours under different PH conditions. The asterisk (*P < .05, **P < .01) suggested that the differences were statistically significant.

Duckweed treated with wild type and 50 μM CdCl_2_ were treated with different pH (4.3, 5.3, 5.8, 6.3, 7.3) for 24 hours. NO was labeled with specific NO fluorescence probe DAF-FM, and the rhizome was photographed by the fluorescence microscope. Figure 4 showed the fluorescence intensity of NO dyes under different pH values. The study showed that, with the pH value increasing from 4.3 to 6.3, endogenous NO levels increased significantly at pH 5.3 and 7.3 under the stress of Cd, and the highest endogenous NO levels were detected in the rhizome at pH 5.3. However, in the control group, endogenous NO levels were significantly lower at pH 4.3 than at other pH levels. It was found that the endogenous NO level at pH 5.3 reached its highest value under the stress of Cd. This indicates that the endogenic NO produced by the rhizome of duckweed under cadmium stress may be influenced by pH.

### 3.6 Differential expression of genes in photosynthetic system between NHX1 transgenic duckweed and WT duckweed

Under Cd stress, differential gene expression related to photosynthesis in NHX1 transgenic duckweed compared to WT duckweed was measured. As shown in Table 4, genes of photosynthesis were analyzed. In genes of photosynthesis, photosystem II oxygen-evolving enhancer protein 3 was the most significantly down-regulated gene, which has fallen 2.4646 log_2_ Fold Change. There are also a few up-regulated genes, such as phosphoenolpyruvate carboxykinase (ATP) [EC:4.1.1.49], malate dehydrogenase (oxaloacetate-decarboxylating) (NADP^+^) [EC:1.1.1.40] and photosystem II oxygen-evolving enhancer protein 2, respectively, which has raised 5.14, 7.19 and 1.78 log_2_ Fold Change. The above results indicate that the photosynthetic capacity of duckweed was significantly increased under cadmium stress (50 μM) in NHX1 transgenic duckweed.

**Table 4.** Changes in gene expression levels related to photosynthesis. (OE-Cd vs WT-Cd)

Description	Gene ID	OE_CdReadcount	WT_CdReadcount	log2FoldChange	pval	padj
phosphoenolpyruvate carboxykinase (ATP) [EC:4.1.1.49]	Cluster-2695.0	17.32	0.48	5.14	0.00	0.01
malate dehydrogenase (oxaloacetate-decarboxylating) (NADP+) [EC:1.1.1.40]	Cluster-7365.101961	26.23	0.00	7.19	0.00	0.02
photosystem II oxygen-evolving enhancer protein 3	Cluster-7365.50996	140.48	777.08	−2.46	0.00	0.00
photosystem I subunit III	Cluster-7365.55496	584.73	1337.66	−1.19	0.00	0.00
photosystem II oxygen-evolving enhancer protein 2	Cluster-7365.49749	85.70	24.90	1.78	0.00	0.00

## 4. Discussion

NO, an important signal molecule in the planting object, is widely involved in the sprout, development, flowering, aging, and abiotic stress responses of plants.^15^ NO is a vital part in abiotic stress, for example, drought stress, salt stress, and heavy metal stress.^21^

In our experiment, endogenous NO of WT duckweed accumulated in large quantities under the stress of Cd, and showed a trend of first rising and then decreasing (Figure 2). Compared with CK, WT duckweed under cadmium stress for 24 hours showed a significant decrease, indicating that NO is an important part of combating cadmium stress. In previous studies, it has been proved that Cd stress can cause a lack of Ca and the excessive production of ROS, thus leading to a large reduction of NO,^19^ which showed same variation trend as our study (Figure 1). Cd^2+^ and Ca^2+^ have a competitive relationship of transporter protein,^22^ intracellular and plasma membrane Ca^2+^ binding protein, resulting in the reduction of Ca. However, with the decrease of Cd accumulation in NHX1 transgenic duckweed, the Ca level was increased in NHX1 transgenic duckweed compared to WT duckweed during Cd stress (Figure 4). Ca^2+^ has a negative effect on the production of O_2_^−^, resulting in the excessive production of ROS. At the same time, Cd stress can also cause the decrease of SOD, CAT, and other antioxidants, so that ROS can’t be removed in time, resulting in the accumulation, which further leads to the reduction of NO and a substantial reduction.

At the same time, our experimental results showed that endogenous NO level increased after 24 h of Cd treatment in NHX1 transgenic duckweed, and significantly increased when amiloride inhibitor was added. In our previous studies, it was found that the overexpression of NHX1 promoted the outflow of Cd^2+^ and the internal flow of H^+^, thereby reducing the internal pH value, and then the H_2_O_2_ burst, which strengthens the activities of SOD and POD enzymes.^12^ Therefore, we speculated that the pH change caused by the overexpression of NHX1 lead to an increase in endogenous NO content. In order to confirm this speculation, our experiment changed the pH of wild-type duckweed under cadmium stress, and found that endogenous NO level of WT duckweed reached the highest value at a pH of 5.3, when the pH was lower than the normal pH of 5.8, which just verified this speculation. and this could also cause an increase of endogenous NO level.

The two main sources of NO production in plant cells are arginine-dependent pathways^23^ and nitrite-dependent pathways^13, 14^ and dependent on nitrate reductase (NR) and nitric oxide synthase (NOS). In transcriptome analysis of the key enzyme for NO synthesis, the expression level of nitrate reductase gene decreased under cadmium stress for 24 h, which may explain the observed reduction of NO after treating duckweed with Cd. In transcriptome analysis of calmodulin expression, after 24 h of cadmium treatment, calmodulin expression in OE duckweed was lower, while calmodulin-binding transcription activator and calmodulin-binding receptor were increased, indicating that Ca^2+^ was regulated in OE duckweed under cadmium stress. Previous experimental studies have shown that Ca^2+^/CaM may regulate the production of NO by regulating the activity of NOS, and the reduction of Ca^2+^/CaM may lead to the reduction of NO (Jeandroz *et al*., ^24^2013). This can also explain the decreased endogenous NO of WT duckweed under the stress of Cd.

## 5. Conclusion

In summary, the results of this work report provide clear evidence that the NHX1 gene influences the endogenous NO level of duckweed to improve tolerance under Cd treatment. Moreover, the expression of the Nitrate reductase gene was down-regulated and NO synthesis was decreased under Cd stress in a pH-dependent manner. And this Cd tolerance effect in NHX1 duckweed is regulated in different ways, specifically in the effects on pH value, calmodulin, and key enzymes for NO synthesis. On the other hand, the in-depth study of the molecular mechanism of NHX1 gene and NO signal in the process of resisting Cd stress will also help us to explore more effective ways to resist heavy metal pollution.

## References

[1] Di Toro DM, Mahony JD, Hansen DJ, Scott KJ, Hicks MB, Mayr SM, Redmond MS. Toxicity of cadmium in sediments: the role of acid volatile sulfide. Environ Toxicol Chem. 1990;9(12):1487–7. doi:10.1002/etc.5620091208.

[2] Cheng M, Wang A, Tang C. Ammonium-based fertilizers enhance Cd accumulation in Carpobrotus rossii grown in two soils differing in pH. Chemosphere. 2017;188:689–696. doi:10.1016/j.chemosphere.2017.09.032. 28923732

[3] Huang H, Li M, Rizwan M, Dai Z, Yuan Y, Hossain MM, Cao M, Xiong S, Tu S. Synergistic effect of silicon and selenium on the alleviation of cadmium toxicity in rice plants.Journal of. Hazardous Mat. 2021;401:123393. doi:10.1016/j.jhazmat.2020.123393. 32763692

[4] Makino T, Sugahara K, Sakurai Y, Takano H, Kamiya T, Sasaki K, Itou T, Sekiya N. Remediation of cadmium contamination in paddy soils by washing with chemicals: selection of washing chemicals. Environ Pollut. 2006;144(1):2–10. doi:10.1016/j.envpol.2006.01.017. 16580105

[5] Neill S, Barros R, Bright J, Desikan R, Hancock J, Harrison J, Morris P, Ribeiro D, Wilson I. Nitric oxide, stomatal closure, and abiotic stress. J Exp Bot. 2008;59(2):165–176. doi:10.1093/jxb/erm293. 18332225

[6] Santisree P, Bhatnagar-Mathur P, Sharma KK. NO to drought-multifunctional role of nitric oxide in plant drought: do we have all the answers? Plant Sci. 2015;239:44–55. doi:10.1016/j.plantsci.2015.07.012. 26398790

[7] Guo Y, Tian Z, Yan D, Zhang J, Qin P. Effects of nitric oxide on salt stress tolerance in Kosteletzkya virginica. J NE Fores Univ. 2009;37(7):62–64. doi:10.3969/j.1000-5382.2009.07.021.

[8] Zhang Y, Wang L, Liu Y, Zhang Q, Wei Q, Zhang W. Nitric oxide enhances salt tolerance in maize seedlings through increasing activities of proton-pump and Na+/H+ antiport in the tonoplast. Planta. 2006;224(3):545–555. doi:10.1007/s00425-006-0242-z. 16501990

[9] Xu J, Wang W, Yin H, Liu X, Sun H, Mi Q. Exogenous nitric oxide improves antioxidative capacity and reduces auxin degradation in roots of Medicago truncatula seedlings under cadmium stress. Plant Soil.2010. 326:321–330. doi: 10.1007/s11104-009-0011-4.

[10] Yang L, Han Y, Wu D, Yong W, Liu M, Wang S, Liu W, Lu M, Wei Y, Sun J. Salt and cadmium stress tolerance caused by overexpression of the Glycine Max Na+/H+ Antiporter (GmNHX1) gene in duckweed (*Lemna turionifera* 5511). Aquat Toxicol. 2017;192:127–135. doi:10.1016/j.aquatox.2017.08.010. 28946066

[11] Barragán V, Leidi EO, Andrés Z, Rubio L, De Luca A, Fernández JA, Cubero B, Pardo JM. Ion Exchangers NHX1 and NHX2 mediate active potassium uptake into vacuoles to regulate cell turgor and stomatal function in *arabidopsis*. Plant Cell. 2012;24(3):1127–1142. doi:10.1105/tpc.111.095273. 22438021 PMC3336136

[12] Yang L, Wei Y, Li N, Zeng J, Han Y, Zuo Z, Wang S, Zhu Y, Zhang Y, Sun J, et al. Declined cadmium accumulation in Na+/H+ antiporter (NHX1) transgenic duckweed under cadmium stress. Ecotoxicol Environ Saf. 2019;182:109397. doi:10.1016/j.ecoenv.2019.109397. 31299476

[13] Stöhr C, Strube F, Marx G, Ullrich WR, Rockel P. A plasma membrane-bound enzyme of tobacco roots catalyses the formation of nitric oxide from nitrite. Planta. 2001;212(5–6):835–841. doi:10.1007/s004250000447. 11346959

[14] Xu J, Wang W, Yin H, Liu X, Sun H, Mi Q. Exogenous nitric oxide improves antioxidative capacity and reduces auxin degradation in roots of Medicago truncatula seedlings under cadmium stress. Plant Soil. 2010;326(1–2):321–330. doi:10.1007/s11104-009-0011-4.

[15] Ahmad P, Ahanger MA, Alyemeni MN, Wijaya L, Alam P. Exogenous application of nitric oxide modulates osmolyte metabolism, antioxidants, enzymes of ascorbate-glutathione cycle and promotes growth under cadmium stress in tomato. Protoplasma. 2018;255(1):79–93. doi:10.1007/s00709-017-1132-x. 28643085

[16] Hoffmann H, Frieler K, Schlattmann P, Hamm B, Dewey M Influence of statin treatment on coronary atherosclerosis visualised using multidetector computed tomography. Eur Radiol. 2010;20:2824–2833. doi:10.1007/s00330-010-1880-x. 20640900

[17] Rodriguez-Serrano M, Romero-Puertas MC, Zabalza A, Corpas FJ, Gomez M, Del Rio LA, Sandalio LM. Cadmium effect on oxidative metabolism of pea (Pisum sativum L.) roots. Imaging of reactive oxygen species and nitric oxide accumulation in vivo. Plant Cell Environ. 2006;29:1532–1544. 10.1111/j.1365-3040.2006.01531.x. 16898016

[18] Jedelská T, Luhová L, Petřivalský M. Nitric oxide signalling in plant interactions with pathogenic fungi and oomycetes. J Exp Bot. 2021;72(3):848–863. doi:10.1093/jxb/eraa596. 33367760

[19] Correa-Aragunde N, Foresi N, Lamattina L. Nitric oxide is a ubiquitous signal for maintaining redox balance in plant cells: regulation of ascorbate peroxidase as a case study. J Exp Bot. 2015;66(10):2913–2921. doi:10.1093/jxb/erv073. 25750426

[20] Mishra V, Singh P, Tripathi DK, Corpas FJ, Singh VP. Nitric oxide and hydrogen sulfide: an indispensable combination for plant functioning. Trends Plant Sci. 2021;26(12):1270–1285. doi:10.1016/j.tplants.2021.07.016. 34417078

[21] Rivetta A, Negrini N, Cocucci M. Involvement of Ca2+-calmodulin in Cd2+ toxicity during the early phases of radish (*Raphanus sativus L*.) seed germination. Plant Cell Environ. 1997;20(5):600–608. doi:10.1111/j.1365-3040.1997.00072.x.

[22] Clemens S. Toxic metal accumulation, responses to exposure and mechanisms of tolerance in plants. Biochimie. 2006;88(11):1707–1719. doi:10.1016/j.biochi.2006.07.003. 16914250

[23] Corpas FJ, Barroso JB, Carreras A, Valderrama R, Palma JM, León AM, Sandalio LM, Del Río LA. Constitutive arginine-dependent nitric oxide synthase activity in different organs of pea seedlings during plant development. Planta. 2006;224(2):246–254. doi:10.1007/s00425-005-0205-9. 16397797

[24] Jeandroz S, Lamotte O, Astier J, Rasul S, Trapet P, Besson-Bard A, Bourque S, Nicolas-Francès V, Ma W, Berkowitz GA, et al. There’s More to the Picture Than Meets the Eye: Nitric Oxide Cross Talk with Ca2+ Signaling. Plant Physiol. 2013;163:459–470. 10.1104/pp.113.220624. 23749853 PMC3793028

